# The effects of oral administration of the novel muscarinic receptor antagonist DA-8010 on overactive bladder in rat with bladder outlet obstruction

**DOI:** 10.1186/s12894-020-00611-8

**Published:** 2020-04-17

**Authors:** Jin Bong Choi, Seung Hwan Jeon, Eun Bi Kwon, Woong Jin Bae, Hyuk Jin Cho, U-Syn Ha, Sung-Hoo Hong, Ji Youl Lee, Sae Woong Kim

**Affiliations:** 1 Department of Urology, Bucheon St. Mary’s Hospital, College of Medicine, The Catholic University of Korea, Seoul, Republic of Korea; 2 Department of Urology, Seoul St. Mary’s Hospital, College of Medicine, The Catholic University of Korea, Seoul, Republic of Korea; 3 Korea Bio Medical Science Institute, Seoul, Republic of Korea; 4 Department of Urology, Seoul St. Mary’s Hospital, College of Medicine, The Catholic University of Korea, Seoul, Republic of Korea and Catholic Integrative Medicine Research Institute, The Catholic University of Korea, Banpo-daero 222, Seocho-gu, Seoul 06591 Seoul, Republic of Korea

**Keywords:** Bladder outlet obstruction, Muscarinic antagonists, Overactive bladder

## Abstract

**Background:**

DA-8010 is a novel compound developed for the treatment of overactive bladder (OAB) and urinary incontinence. The aims of this study were to investigate the effects of DA-8010 on OAB in a rat model.

**Methods:**

Study animals were divided into the following five groups of seven animals each: a sham-operated control group, a control group with partial bladder outlet obstruction (BOO) (OAB group), and three DA-8010 (doses of 0.3 mg/kg/day, 1 mg/kg/day, and 3 mg/kg/day, respectively) with partial BOO groups. Oral administration of the drugs was continued for 14 days after 2 weeks of partial BOO. After 4 weeks of partial BOO, cystometrography was performed in all groups. Additionally, pro-inflammatory cytokines, Rho-kinases, and histology of the bladder were analyzed.

**Results:**

There was a significant increase in the contraction interval and a decrease in contraction pressure in the 3 mg/kg/day DA-8010 group versus those in the OAB group. Rho kinase was also significantly decreased in the DA-8010 3 mg/kg/day dosage treatment group. The increased ratio of collagen to smooth muscle after partial BOO was significantly attenuated in the DA-8010 3 mg/kg/day dosage group.

**Conclusions:**

Oral administration of DA-8010 at 3 mg/kg/day improved findings in an OAB rat model induced by partial BOO. Our results suggest that the novel muscarinic receptor antagonist DA-8010 may be a promising drug for treating patients with OAB.

## Background

Overactive bladder (OAB), a syndrome characterized by urgency (with or without urge incontinence), frequency, and nocturia, is common in both men and women and increases in prevalence with age [1]. Because the mean age of the population worldwide is increasing, OAB is causing an increase in socioeconomic costs and has adverse effects on quality of life [2].

Treatment with antimuscarinic and behavioral therapy is one of the first-line management methods for patients with OAB. These drugs inhibit the binding of acetylcholine to the muscarinic receptor in the detrusor muscle and urothelium during detrusor contraction. Examples of these drugs include oxybutynin, tolterodine, trospium, darifenacin, solifenacin, fesoterodine, propiverine, and flavoxate. While anticholinergics can be effective in OAB, they also carry risk of various complications, such as dry mouth, constipation, acute urinary retention, and visual disturbance. Furthermore, long-term use also increases the risk of psychological problems [3–5]. Therefore, there is a need for new OAB treatments that demonstrate high efficacy and fewer side effects.

DA-8010 ((R)-(1-methylpyrrolidin-3-yl) methyl (3′-chloro-4′-fluoro-[1,1′-biphenyl]-2-yl)carbamate) is a novel compound that has been developed by the Dong-A ST Pharmaceutical Company (Yongin, Korea) for the treatment of OAB and urinary incontinence [6]. DA-8010 is a highly potent M_3_ antagonist and has appeared more highly selective for the urinary bladder over the salivary glands, large intestine, and heart in preclinical studies compared with other antimuscarinic agents [6]. The aim of this study was to investigate the effects of DA-8010 on OAB in a rat model.

## Methods

### Experimental animals and treatment

Male Sprague-Dawley rats weighing 250 g to 300 g were obtained from Orient Bio Co., Ltd. (Seongnam, Kyeonggi, South Korea) and were randomly divided into five groups of seven animals each. The rats were fed standard rat food and had free access to food and water in an animal room maintained at a constant temperature (18–22 °C) and humidity (40–60%) with a 12-h light/dark cycle. These groups were (1) a sham-operated control group, (2) a partial bladder outlet obstruction (BOO) control group (OAB group), (3) a DA-8010 (0.3 mg/kg/day) with partial BOO group, (4) a DA-8010 (1 mg/kg/day) with partial BOO group, and (5) a DA-8010 (3 mg/kg/day) with partial BOO group. The drugs, which were dissolved in 1 ml of distilled water, were orally administered once a day for 14 days at 2 weeks after BOO in each group.

### Transperitoneal ligation of the urethra

As stated, our OAB rat model was induced by partial BOO. This method has been widely used for the analysis of detrusor overactivity (DO) in rat models. In this study, partial BOO was surgically induced through a transperitoneal approach, as previously described [7, 8]. A 25-gauge polyethylene tube was placed on top of the urethral-vesical junction and ligated using 3–0 silk with similar forces in all rats. The tube was then removed to create a partial BOO.

### Cystometrography

Cystometrography was performed in all groups with the animals in an anesthetized state after 2 weeks of oral drug administration. After exposure of the bladder by a midline lower abdominal incision, a 25-gauge needle connected to a polyethylene tube was inserted through the bladder dome. The polyethylene tube was connected to a pressure transducer and a syringe pump via 3-way stopcock [9]. Saline was infused at a rate of 0.04 mL/min after emptying the bladder, and the nonvoiding contractions during the filling phase of cystometry were measured. The contraction interval and detrusor pressure were recorded on a polygraph through the use of a pressure recording catheter. Uninhibited detrusor contraction was assessed during the 2 min from 4 min before voiding contraction, and contractions were defined by an amplitude higher than 4 cm of H_2_O from baseline pressure [10]. At the end of the cystometrography, the rats were sacrificed under sodium pentobarbital anesthesia according to the Guide for the Care and Use of Laboratory Animals [11].

### Western blot analysis

After bladder tissue was ground into powder, the protein was extracted through cell lysis and quantified with the Thermo Scientific Pierce BCA Protein Assay Kit (Thermo Fisher Scientific Waltham, MA, USA). Briefly, 60 μg of quantitative proteins were boiled in loading buffer containing sodium dodecyl sulfate, 100 mM of dithiothreitol, 0.01% bromophenol blue, and 62.6 mM of Tris hydrochloride adjusted to pH 6.8. After sodium dodecyl sulfate polyacrylamide gel electrophoresis, the proteins were moved to Hybond® ECL™ nitrocellulose membranes (Amersham Biosciences, Little Chalfont, UK). The membranes were blocked with 5% nonfat milk in Tris-buffered saline containing 0.1% Tween® 20 (Sigma-Aldrich, St. Louis, MO, USA). Next, the membranes were probed with antibodies against RhoA, Rho-associated protein kinase (ROCK)-I, ROCK-II (BD Pharmingen, San Diego, CA, USA), and β-actin (Santa Cruz Biotechnology, Dallas, TX, USA). After the membranes were washed, they were probed with horseradish peroxidase secondary antibodies (Santa Cruz Biotechnology, Dallas, TX, USA). Expression levels were measured by the density of the bands using a luminescent image analyzer (Fujifilm, Tokyo, Japan) [12].

### Cytokine analyses

To investigate the anti-inflammatory effects of the treatment, we analyzed the levels of interleukin (IL)-6 and IL-8 cytokines. The supernatant was obtained via centrifugation of the blood for 10 min at 3000 rpm. The cytokine concentration was measured using a spectrophotometer with an enzyme-linked immunosorbent assay kit (R&D Systems, Minneapolis, MN, USA) [13].

### Histologic analysis

Bladder tissue samples were fixed with 4% paraformaldehyde for 1 day and then embedded in paraffin. After that, slice sections were made for Masson’s trichrome staining for bladder tissue observation. The color distribution was measured using Image-Pro® Plus (Media Cybernetics Inc., Rockville, MD, USA), and the ratio of collagen to smooth muscle was calculated [14].

### Statistical analysis

All data are expressed as the mean ± standard error. Statistical analysis was conducted using SPSS version 22.0 software (IBM Corp., Armonk, NY, USA). Experimental groups were compared using analysis of variance with Tukey’s multiple comparison test for post hoc analysis, and *P* values < 0.05 were considered significant**.**

## Results

### Cystometrography

The contraction intervals and the contraction pressure in the 3 mg/kg/day DA-8010 group were significantly different from those in the OAB group (Table 1). After 2 weeks of oral medication, the contraction pressure of the OAB group was significantly greater than that of the control group (*P* < 0.01). Additionally, the contraction pressure of the 3 mg/kg/day DA-8010 group was significantly lower than that of the OAB group (*P* < 0.05). Reductions in contraction pressure were also observed in the 0.3 mg/kg/day and 1 mg/kg/day DA-8010 groups, but these changes were not statistically significant. The contraction interval of the OAB group was significantly lower than that of the control group (*P* < 0.01), while the contraction interval of the 3 mg/kg/day DA-8010 group was significantly greater than that of the OAB group (*P* < 0.05). Increases in the contraction interval were also observed in the other DA-8010 (i.e., the 0.3 mg/kg/day and 1 mg/kg/day dosage) groups, but these changes were not statistically significant.

**Table 1:** The contraction intervals and the contraction pressure during continuous infusion cystometry

Parameters	Control	OAB	DA-8010 (0.3 mg)	DA-8010 (1 mg)	DA-8010 (3 mg)
Contraction interval (seconds)	60.81 ± 14.84	6.97 ± 2.95^a^	9.22 ± 4.03	20.89 ± 8.31	22.51 ± 10.89^b^
Contraction pressure (cmH_2_O)	34.35 ± 11.83	51.83 ± 17.95^a^	46.81 ± 16.38	42.09 ± 15.59	38.82 ± 17.31^b^

Data are presented as the mean ± standard error

*Abbreviations*: *OAB* overactive bladder

^a^*P* value < 0.01, OAB group vs. control group

^b^*P* value < 0.05, DA-8010 (3 mg) group vs. OAB group

### Western blot analysis

**Fig. 1:**
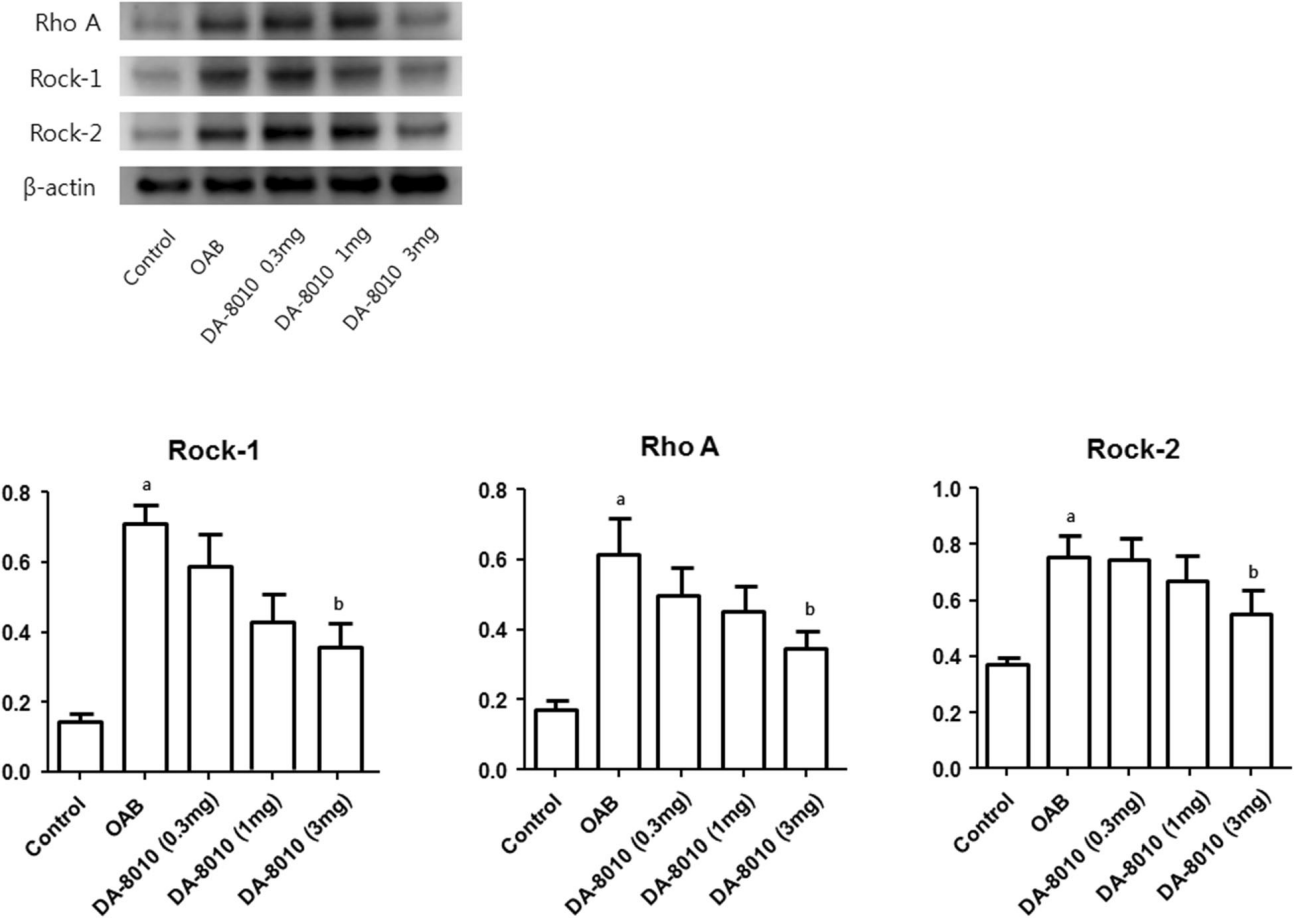
Comparison of RhoA, ROCK-I, and ROCK-II. ^a^*P* < 0.01 compared with the control group; ^b^*P* < 0.05 compared with the OAB group

The OAB group showed significantly higher expression of RhoA, ROCK-I, and ROCK-II in the bladder than did the control group (*P* < 0.01). These levels were significantly lower in the 3 mg/kg/day DA-8010 group than in the OAB group (*P* < 0.05). However, there were no statistically significant changes in the other DA-8010 groups receiving 0.3 mg/kg/day or 1 mg/kg/day dosage (Fig. 1).

### Pro-inflammatory cytokine levels

**Fig. 2:**
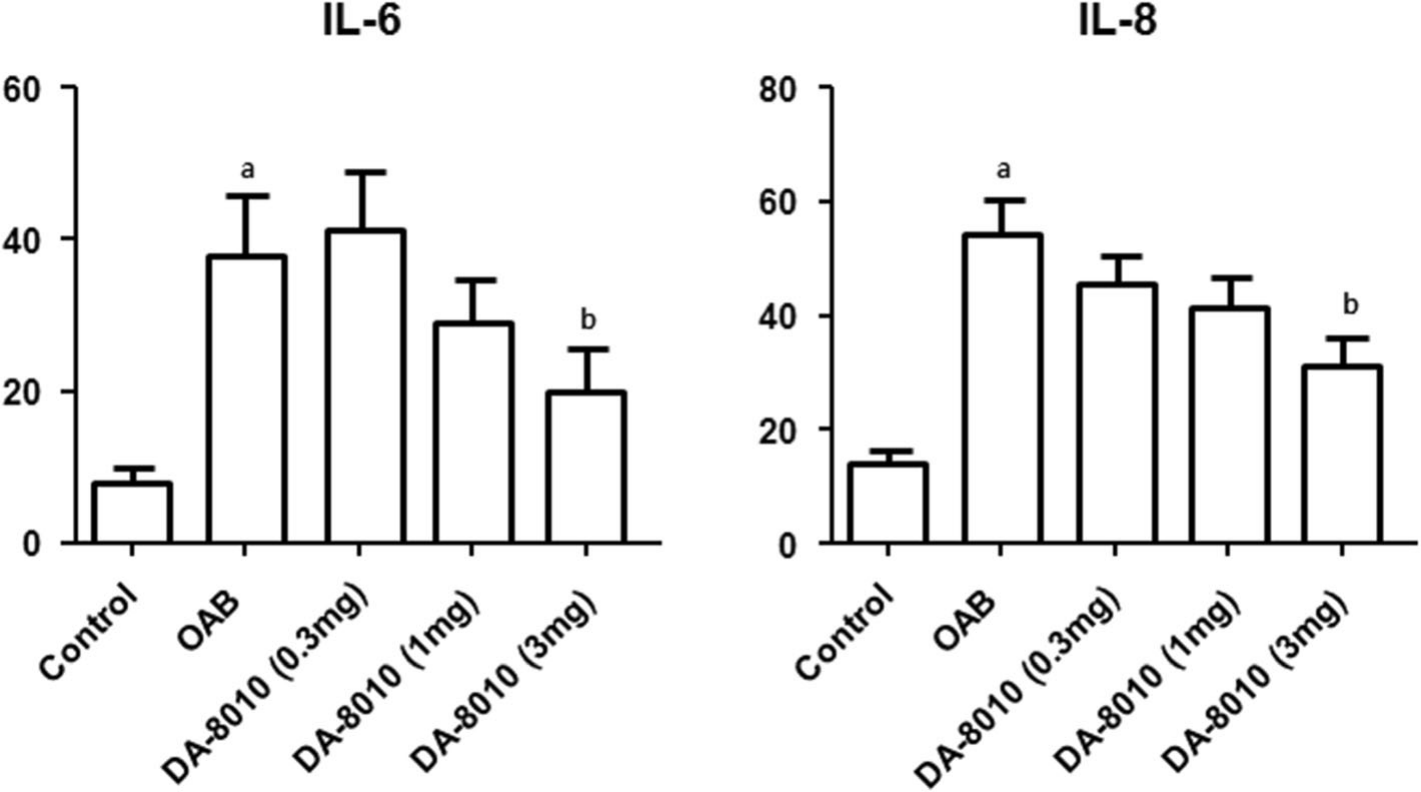
Comparison of cytokines. ^a^*P* < 0.01 compared with the control group; ^b^*P* < 0.05 compared with the OAB group

Significantly higher levels of IL-6 and IL-8 were noted in the OAB group than in the control group (*P* < 0.01). After administration of 3 mg/kg/day of DA-8010, a significant decrease in IL-6 and IL-8 levels was observed compared with those in the OAB group (*P* < 0.05). There were no statistically significant changes in the DA-8010 groups receiving 0.3 mg/kg/day or 1 mg/kg/day (Fig. 2).

### Histologic analysis

**Fig. 3:**
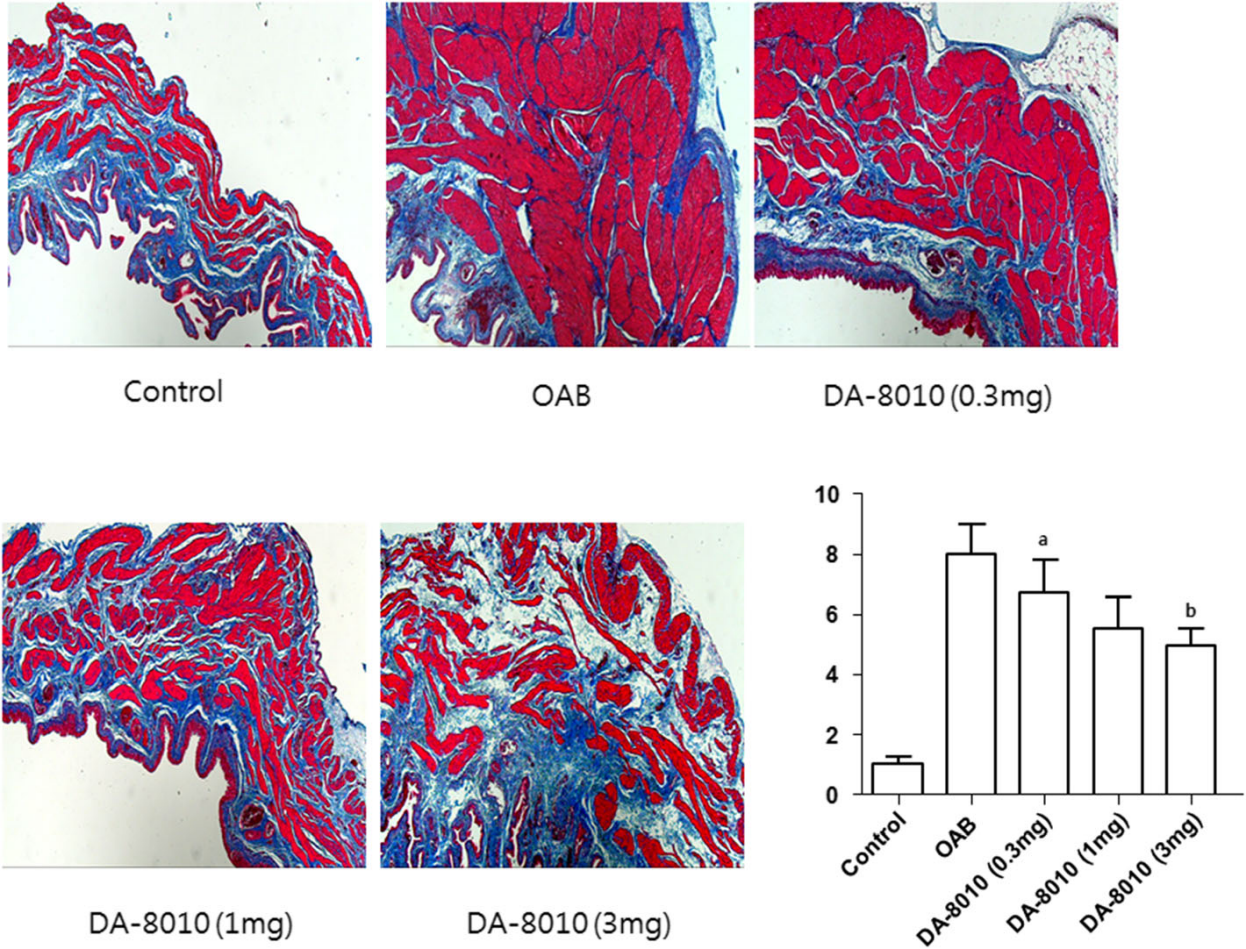
Comparison of histologic findings and the ratio of collagen to smooth muscle among the five groups. ^a^*P* < 0.01 compared with the control group; ^b^*P* < 0.05 compared with the OAB group

The ratio of collagen to smooth muscle identified by image analysis was higher in the OAB group than in the control group, indicating increased bladder fibrosis. However, after 3 mg/kg/day DA-8010 treatment, this increased ratio was attenuated significantly (*P* < 0.05). There were no statistically significant changes in either the 0.3 mg/kg/day DA-8010 group or the 1 mg/kg/day DA-8010 group (Fig. 3).

## Discussion

M_3_ muscarinic receptors are well known to play a predominant role in mediating bladder muscle, although both the M_2_ and M_3_ muscarinic receptor subtypes are located on bladder smooth muscle [15, 16]. DA-8010 is a highly potent M_3_ antagonist with a high binding affinity for the human M_3_ muscarinic receptor, with a pKi of 8.81 ± 0.05, and is more highly selective for the urinary bladder over the salivary glands compared with other antimuscarinic agents. Intravenous single-dose administration of DA-8010 (0.03 mg/kg and 0.1 mg/kg) demonstrated beneficial effects on the DO induced by partial BOO in conscious rats, with a significant increase in micturition intervals and micturition volume [17]. Therefore, the high potency and selectivity of DA-8010 are expected to provide therapeutic benefit with a lesser frequency/degree of side effects than that observed with other antimuscarinic agents. We observed the functional efficacy of DA-8010 on OAB in a rat model in this study. The main findings were as follows: (1) there was a significant increase in contraction interval and a decrease in contraction pressure in the 3 mg/kg/day DA-8010 group, and (2) the increased ratio of collagen to smooth muscle after partial BOO was significantly attenuated in the 3 mg/kg/day DA-8010 group.

Because of the legal and ethical problems associated with using human materials for research, much of our understanding of human voiding function has come from research using animal models [18]. In particular, BOO in humans can be surgically replicated in animal models. Experimental partial BOO in rats is known to increase bladder weight and alter voiding patterns, with changes in central and peripheral nerve control [19]. The most common method to prompt OAB in rats is by inducing partial BOO by silk ligation around the urethra through a transperitoneal approach. In previous reports, bladder pressures, maximal voiding pressure, and frequency of nonvoiding contractions started to increase after 2 weeks of partial BOO using this method [20, 21].

The present study revealed differences in Rho-kinase activity between the OAB group and DA-8010 treatment groups. Rho-kinase was discovered in 1996 by Amano et al. [22]. Kureishi et al. reported that Rho-kinase modulates smooth muscle contraction directly through myosin light-chain phosphorylation without increasing the intracellular Ca^2+^ concentration [23]. Rho-kinase is activated by RhoA GTPase, and RhoA is regulated by guanine nucleotide exchange factors (GEFs), GTPase-activating proteins (GAPs), and guanine nucleotide dissociation inhibitors (GDIs). GEFs are activated by cell stimulation, including that of the muscarinic acetylcholine receptor [24], especially the muscarinic acetylcholine M_3_ receptor, which activates phospholipase C, stimulates RhoA GTPase activity and induces bladder contraction directly [25]. Therefore, the novel muscarinic receptor antagonist DA-8010 could decrease the expression of RhoA, ROCK-I, and ROCK-II in the bladder.

Pro-inflammatory cytokines such as IL-6 and IL-8 have been reported to be increased in bladders with DO induced by BOO [12, 26]. A clinical study reported that solifenacin improves inflammation by decreasing the levels of inflammatory cytokines [27]. Our results showed that these mechanisms are similar to those of DA-8010. In addition, the inflammatory response due to partial BOO can eventually produce decompensation to fibrosis [28]. Our histologic analysis also revealed that DA-8010 decreased bladder fibrosis significantly compared to BOO alone. These results suggest that DA-8010, by decreasing the inflammatory responses, may have an anti-fibrotic effect.

However, our study has some limitations. First, although we ligated the urethra-vesical junction with similar forces in all rats to produce partial BOO, dominant DO might not have been formed in all rats. Second, changes in bladder function after partial BOO vary with time. Maciejewski et al. reported on urodynamic outcomes in a partial BOO rat model. Outcomes were assessed after four, eight, 12, and 16 weeks, with results indicating an initial increase in bladder capacity and compliance at 4 weeks, followed by a steady decline [21]. Our study investigated urodynamic findings after only 4 weeks of partial BOO. For this reason, we were unable to quantify changes in the urodynamic findings at different stages during the experimental period. Thus, long-term and more detailed preclinical studies are needed.

## Conclusions

OAB is a chronic disease that not only lowers quality of life but is also difficult to treat, although it is not life-threatening. In this study, administration of a 3 mg/kg/day dose of DA-8010 improved the findings of DO induced by partial BOO in a rat model. Our results suggest that the novel muscarinic receptor antagonist DA-8010 could be a promising option for treating OAB in patients.

## Data Availability

The datasets used in this study are available from the corresponding author on reasonable request.

## Abbreviations

OAB: Overactive bladder
BOO: Bladder outlet obstruction;
DO: Detrusor overactivity
ROCK: Rho-associated protein kinase
IL: Interleukin
